# Mindful Learning Improves Positive Feelings of Cancer Patients’ Family Caregivers

**DOI:** 10.3390/ijerph16020248

**Published:** 2019-01-16

**Authors:** Liuna Geng, Jian Wang, Liping Cheng, Binbin Zhang, Hui Shen

**Affiliations:** School of Social and Behavioral Sciences, Nanjing University, Nanjing 210023, China; dr.jianwang@smail.nju.edu.cn (J.W.); chenglp@ahyjjh.com.cn (L.C.); zhangbinbin10@countrygarden.com.cn (B.Z.); shenhui@nju.edu.cn (H.S.)

**Keywords:** mindfulness, positive feelings, family caregivers, cancer patients

## Abstract

Positive feelings are an important health dimension for family caregivers of cancer patients. The aim of this study was to investigate whether Langerian mindfulness is a valid proactive method to increase the positive feelings of family caregivers for cancer patients. Participants were randomly assigned to either a mindfulness group or a mindlessness group and completed the Caregiver Reaction Assessment (CRA) as a measure of caregivers’ feelings before the intervention. Subsequently, both groups were given four sessions of mindfulness training using “innovation classification”. Finally, participants completed the Langer Mindfulness Scale (LMS) and the Positive Aspects of Caregiving (PAC) scale as post-intervention measures. The results revealed that participants in the mindfulness and mindlessness groups differed significantly in LMS and PAC scores, with the mindfulness group having higher levels of positive feelings than those in the mindlessness group. The results also indicated that mindfulness level significantly predicted positive feelings of caregivers. Thus mindful interventions may play a meaningful role in promoting family caregivers’ spirituality and faith, improving the willingness of sharing their thoughts, beliefs, and grief, which could be useful for increasing the positive feelings of caregivers.

## 1. Introduction

Incidences of cancer in the world have been rising, with the World Health Organization listing cancer as a chronic disease of high intensity and sustained care needs. Family caregivers are critically important to provide care and enhance the well-being of patients with cancer [1]. Most previous research has indicated that family caregivers experience substantial psychological stress and depression [2]. However, as the focus has been on negative effects on family caregivers, the positive effects of caregiving have been overlooked [3]. This study aimed to investigate family caregivers’ positive feelings and salutary benefits that can arise from the caregiving process.

The majority of research on cancer caregiving has long focused on stress, anxiety, and depression [4]. Researchers have pointed out that family caregivers often experience time constraints, financial hardship, and poor communication with health professionals [5]. Research has revealed that economic and mental pressure exerts a significant impact on quality of life in cancer family caregivers [6]. However, some researchers have focused on family caregivers’ positive experiences when the terminal patient is still alive and post-traumatic growth [7]. Moreover, the relationship between patients and family caregivers has been found to become more intimate [8]. A growing number of studies have examined positive outcomes and found that caregivers typically provide better care when they perceive the caregiving experience as satisfying and rewarding [9]. In general, benefits and rewards from cancer caregiving are equally valued and indispensable to family caregivers as are losses and burdens [10].

Interventions regarding caregivers’ negative emotions are more common than those targeting positive emotions, for example, the solutions and connections intervention, based on theories of stress coping [11], and especially, cognitive-behavioral interventions applied to reduce caregivers’ depression by increasing participation in pleasant events [12]. Traditionally, few researchers have looked at interventions targeting caregivers’ positive feelings, however, a growing number of researchers have shifted from exclusively investigating family caregivers’ negative feelings. From a psychological perspective, positive aspects of caregivers’ feelings should be paid increasing attention in the future [13]. Therefore, the current study is grounded in the belief that interventions in relation to caregivers’ positive feelings are required. Despite differences in emphasis, it was anticipated that the study of negative emotions could provide a reference for the study of positive emotions.

Interventions targeting positive feelings have increasingly attracted researchers’ attention [14], and some studies have indicated a strong connection between mindfulness and psychological well-being [3]. Mindfulness interventions have been promoted and used as positive interventions for caregivers, and recent innovations in clinical interventions have also seen an increase in the effectiveness of mindfulness approaches [15]. There are main genres in the field of mindfulness interventions. One genre is based on the integration of Buddhist teachings into emotion research by Kabat-Zinn in the late 1970s, who considered that mindfulness refers to consciously non-judgmental attitudes in the present moment [16]. Mindfulness-based stress reduction (MBSR) was developed by Kabat-Zinn and has been applied in research, involving practices such as watching a DVD and practicing yoga to relieve stress and anxiety and has also been used with individuals who suffer from pain and depression. The other genre was introduced into modern society by Langer, who proposed that individuals generate a positive relationship between the propensity to be mindful and liberal thinking styles (e.g., in a traditional mindset condition, people are likely to impose stigma on those with a disability; in contrast, individuals with a physical disability may be considered fit for sitting work from multiple perspectives if in a mindfulness state) [17]. Comparing the two genres, Kabat-Zinn considered that mindfulness should involve paying attention to the present moment, without judgment or consciousness. However, if individuals are not aware that they are not paying attention because of their changing reality, the instruction of paying attention itself may play a smaller role [18]. On the other hand, Langer believed that “being in the moment” was represented by the simple act of noticing new things, as this instruction leads to focusing attention [19]. More importantly, Kabat-Zinn focused on reducing negative emotions, while Langer aimed to promote positive feelings. Despite the common distinction between the two mindfulness approaches, they appear to be highly related one to each other [20]. The present study adopted a Langerian mindfulness approach for the experimental intervention. 

Langerian mindfulness has been used in studies examining the relationship between mindfulness and health-related outcomes in caregivers of patients with amyotrophic lateral sclerosis [13], as well as in caregivers of patients with traumatic brain injury (TBI) [21]. Langer proposed that cultivating a state of mindfulness could act as a protective factor against caregiver burden. The greater the mindfulness level for a caregiver, the higher his/her level of psychological well-being in the caregiving experience over time [13]. The self-report online survey completed by the caregivers showed that mindfulness played an important role in promoting positive feelings. This study provided a good theoretical basis for the present research. Furthermore, ample previous evidence exists demonstrating that Langerian mindfulness interventions have beneficial effects on positive feelings. For example, mindfulness interventions led individuals to consider whether information may or may not be true, and even how to exchange an idea for its opposite, which changed their cognitive state and led them to adjust their feelings [22]. The current study considers the role of mindfulness suggested by Langer and concludes with a number of mindfulness applications. For innovation, the focus was placed on promoting positive feelings of family caregivers through a mindfulness intervention.

Overall, based on previous research, the current study aimed to test the effectiveness of a Langerian mindfulness intervention in promoting positive feelings in family caregivers for cancer patients. It was hypothesized that the mindfulness intervention would increase the level of positive feelings in family caregivers. 

## 2. Materials and Methods 

### 2.1. Participants

A total of 60 caregivers of patients with cancer participated in the study. Participants were randomly assigned to either the mindfulness group (*n* = 31) or the mindlessness group (*n* = 29). Participants were recruited from the Department of Oncology in Gulou Hospital, affiliated to Nanjing University in China, and all of them had cared for a patient for longer than one month. Participants were provided with four sessions of mindfulness training (30 min each session). All subjects gave their informed consent for inclusion before they participated in the study. The study was conducted in accordance with the Declaration of Helsinki, and the protocol was approved by the Ethics Committee of Nanjing University. 

### 2.2. Materials

**Mindfulness Intervention.** The mindfulness intervention used was based on the Langerian mindfulness theory and was designed to create a situation in which participants could attend to a given situation from different perspectives in order to break their traditional mindset. Langerian mindfulness often uses “innovation classification”, which creates a flexible state of mind and leads to a number of different consequences: (a) increased sensitivity to the human environment; (b) increased openness to accept new information; (c) creation of new categories for constructing perceptions; and (d) enhanced awareness of multiple perspectives in problem-solving [17]. The intervention in the current paper was based on Geng’s research, including tasks of categorization, multi-angle thinking, and free association [23]. The experimental intervention involved four sessions; in each of them, participants in the mindfulness group were asked to think and write four answers from different perspectives to a picture-related question, while the mindlessness group was only asked to write one answer. In order to keep the number of answers consistent between the groups, the mindlessness group was asked to answer three additional simple questions, such as the date, their interests, and other issues irrelevant to the picture. On day 1, the participants were shown a picture of an old man in the hospital taking care of his wife and were asked about the advantages and disadvantages of taking care of patients. On day 2, participants were asked to think about ways to relieve patients’ stress and pain. On day 3, participants were asked to answer the following questions, “What are four kinds of support that family caregivers could provide to patients? What are four kinds of support that could not be provided?” On day 4, the participants were asked about positive changes that they had experienced since taking care of patients. By contrast, the additional intervention materials employed in the mindlessness condition included tasks of categorization and multi-angle thinking unrelated to the picture content. 

**Langer Mindfulness Scale.** Langer designed the Langer Mindfulness Scale (LMS) to measure individuals’ level of mindfulness [24]. The LMS items are scored using a 7-point Likert-type scale (1 = strongly disagree, 7 = strongly agree) and higher total scores correspond to an increased propensity of individuals to be mindful. Previous research has reported that the LMS has robust validity through correlations with theoretically relevant individual-difference constructs (i.e., the LMS is correlated with the tendency to entertain multiple perspectives) [25]. In the current research, Cronbach’s alpha for the LMS was 0.81.

**Caregiver Reaction Assessment.** Given et al. [26] originally developed the Caregiver Reaction Assessment (CRA), which includes 5 dimensions and 24 items (e.g., “Caring makes me feel good”). It is designed to assess specific aspects of the caregiving situation, including both positive and negative dimensions of caregivers’ reactions. The scale has been widely used to assess the feelings of caregivers of patients with cancer, stroke, and other chronic diseases. Previous research showed that the CRA had a Cronbach’s alpha of 0.68–0.90, and was correlated with caregivers’ health and impact on their daily schedule [27]. The current study used the Chinese version of the CRA [28]. 

**Positive Aspects of Caregiving Scale.** The Positive Aspects of Caregiving (PAC) scale was developed by Tarlow (2004) [29]. Previous research highlighted the significance of measuring the burden of caregivers of patients with severe mental illness [2]. By contrast, positive aspects of care have been taken into account in recent research [30]. In addition to being conceptually equivalent, the CRA and PAC scales are empirically interrelated. These two questionnaires share a similar pattern of correlations with cancer caregivers’ feelings [10], and it is believed that one could be replaced by the other if needed [31]. Consequently, the CRA was chosen as the baseline test and the PAC as the post-test in this study. 

### 2.3. Procedures

All participants voluntarily participated in the experiment and were tested individually in a private room. Participants provided written consent to participate in the study. They were informed that they were to complete a free-association task; a final inquiry showed that no one had surmised the actual purpose of the study. Participants were randomly assigned to two conditions: Mindlessness and mindfulness. Firstly, all participants were assessed using the self-report CRA scale to obtain baseline measurements; next, participants in each group completed either mindfulness or mindlessness intervention tasks; subsequently, they completed the LMS and PAC scales; finally, participants were thanked and debriefed. 

### 2.4. Statistical Analysis

We used the following steps in the analyses: (i) the data were screened according to the principle of three standard deviations above or below the mean scores, and one outlier was excluded. Consequently, there were 30 participants in the mindfulness group and 29 participants in the mindlessness group. (ii) SPSS 20.0 (SPSS Inc., Chicago, IL, USA) was used to examine the differences between the mindfulness and mindlessness groups before the intervention. (iii) The mindlessness and mindfulness groups were identified in LMS scores after the intervention. (iv) PAC scores were analyzed between the mindfulness and mindlessness groups which showed a statistically significant difference; and (v) the Pearson product-moment correlation coefficient was used to analyze the relationship between the LMS and PAC scores, using the “enter” method of regression analysis to validate the relationship between caregivers’ mindfulness level and the level of positive feelings. To validate the findings, all study team members participated in discussions about the empirical analysis and writing of the manuscript. 

## 3. Results

Descriptive analyses and independent-samples t-tests were performed using SPSS 20.0 to examine the differences between the mindfulness and mindlessness groups. The results showed that the scores of the CRA and PAC were significantly positively correlated (*r* = 0.47, *p* < 0.01). Moreover, there was no significant difference between the two groups in CRA scores (*t* = −1.33, *p* = 0.19, Cohen’s *d* = 0.34; M_mindless_ = 68.17, SD = 5.95 versus M_mindful_ = 66.17, SD = 5.66). As predicted, the mindlessness and mindfulness groups differed significantly in LMS scores after the intervention (*t*(60) = 2.61, *p* < 0.05, Cohen’s *d* = 0.68; M_mindless_ = 97.62, SD = 11.04 versus M_mindful_ = 105.97, SD = 13.41). Therefore, the mindfulness intervention was shown to be effective. Regarding PAC scores, there was a statistically significant difference between the mindlessness and mindfulness groups (*t*(60) = 2.56, *p* < 0.05, Cohen’s *d* = 0.67; M_mindless_= 35.59, SD = 7.89 versus M_mindful_ = 40.30, SD = 6.20).

The Pearson correlation coefficient was used to analyze the relationship between the LMS and PAC scores. The results showed that there was a significant positive correlation between the caregivers’ levels of mindfulness and positive feelings (*r* = 0.37, *p* < 0.01). Furthermore, according to the results of the correlation analysis, taking the level of mindfulness as the independent variable, the level of positive feelings as the dependent variable, and using the “enter” method of regression analysis, it was found that caregivers’ mindfulness level can significantly predict their level of positive feelings (*t*(60) = 2.96, *p* < 0.01).

## 4. Discussion

Previous findings have suggested a correlation between individuals’ mindfulness level and happiness [32]. Additionally, mindfulness has been positively associated with well-being [13] as well as with self-compassion and personality traits [33]. However, there are a lack of clinical applications of mindfulness learning focusing on promoting positive feelings of family caregivers of patients with cancer. Therefore, these results expand the field of vision in this research area. On one hand, the scope of positive emotions is expanded, as exploring caregivers’ positive feelings is an innovation of this study, which not only focuses on happiness but also suggests how mindfulness could contribute to increasing individuals’ well-being, self-esteem, and self-affirmation during the care process [28]. On the other hand, this study deepened the specific operational mind-training method “innovation classification” created by Langer [22]. In contrast with previous studies, the current research focused on cognition style and provided preliminary support for the feasibility, acceptability, and effects of a four-session mindfulness intervention for caregivers. The mindful intervention in this study helped caregivers to self-regulate emotion and enhance their positive feelings. 

Reasons for the differences between the mindfulness and mindlessness groups can be traced back to differences between mindfulness and mindlessness. Mindfulness is a cognitively active state characterized by conscious operation and actively distinguishing things in consciousness, a process that relates to the acceptance of experience [34]. In contrast, in mindlessness, the individual interacts with the environment in a passive way and fails to ask questions about the environment, reconsider preformed categories, and seek new distinctions between stimuli [35]. Directly comparing mindful and mindless learning, mindful activity is more concerned with cognitive processing and flexibility. Hence, in a flexible mindset, the caregivers who received the mindfulness intervention needed to draw new distinctions about the care process and provided various answers to each question, inspiring multi-angle thinking. In this way, caregivers may use different points of view to realize how things changed, which led them to look for positive meanings of caregiving, with a more positive attitude towards disease, an enhanced sense of accomplishment, and an understanding of the meaning of life. In addition, unlike mindlessness, which may be thought to be the “default” state [17] causing caregivers to see events in predetermined ways [36], mindfulness makes individuals aware of different interpretations of a particular event and leads them to actively compare and distinguish the differences between the past and the present. Indeed, after mindfulness training, it was found that caregivers had more positive thinking than traditional ideas toward their surroundings and thought that they could achieve a more positive role in the care process [37]. These experiences increase caregivers’ ability to cope with the challenges of caregiving and develop the capability to accept their own condition. Generally, mindful interventions promote positive thinking and, subsequently, enhance positive feelings.

This research showed that mindful interventions could promote caregivers’ positive feelings, which differs from most previous research focusing on relieving the pressure of caregiving [38]. The field has thus far proceeded in the absence of research on the relationship between positive and negative emotions. Moreover, there is no theoretical rationale that lessening negative emotion will promote positive emotional development. Although research has proved that mindfulness is positively related to the quality of life and negatively related to the level of burden [13], whether positive and negative emotions can be transformed into each other remains untested and thus unsubstantiated. The current study focused on how to help caregivers to enhance their positive feelings even if they experience negative emotion. Moreover, the presence of protective factors like mindfulness may mediate the effects of stressors on caregivers’ mastery, spiritual involvement, and beliefs [7]. In this sense, high levels of mastery control over the interpretation of their life circumstances, spiritual involvement, and beliefs may have provided interpretive resources for the development of a satisfying life narrative despite the experience of stress. Therefore, mindful interventions may play a meaningful role in promoting spirituality and faith, which would be useful in increasing caregivers’ positive feelings. In the long term, mindful interventions could be beneficial for both the patients and the caregivers and contribute to a harmonious atmosphere in the hospital and the family. 

While summarizing the effectiveness of the mindfulness intervention, it is reputed that the use of mindfulness-learning material is an important factor and the content of such material must be conducive to the mindfulness intervention. Considering earlier theories and applications of mindfulness, mindfulness interventions were shown to be effective by using a range of different materials divided into two types: (1) universally applicable but irrelevant to the subject; (2) specifically relevant to the subject [38]. In previous work, domain-irrelevant perspectives of mindfulness interventions mainly stressed a complex transfer of cognitive styles from one domain to other irrelevant areas [30]. For instance, Idusohan-Moizer and his colleagues used domain-irrelevant learning materials based on mindfulness-based cognitive therapy for adults with intellectual disabilities to reduce symptoms of depression and anxiety [39]. On the other hand, researchers can also utilize domain-relevant learning materials and test the direct effects of the mindfulness intervention. Specifically, through domain-relevant learning materials, research has shown that individuals are more likely to concentrate on a deeper acquaintance of subjects in one field [40]. For example, Djikic et al. [40] employed domain-relevant mindfulness-learning materials (i.e., 48 pictures including both old and young individuals). They asked participants to sort the pictures under different standards and found that participants in a mindfulness condition reported less stereotype-activated behaviors towards the elderly than did those in a mindlessness condition. Comparing the two types of intervention materials, it should be pointed out that universal materials are available in a wider range and are easy to operate, but require long-term interventions and are thus inconvenient for achieving faster results. Therefore, this can be described as a method to be applied through practice in everyday life, essentially, in order to promote positive feelings to cope with difficulties. On the other hand, targeted intervention materials have achieved obvious positive effects in short-term interventions. The effects of targeted interventions are therefore timely and their purpose limited. Thus, targeted intervention materials can be used with specific groups experiencing emergencies or crises. Considering that having suffering from cancer is a sudden crisis for caregivers, our research initially tried to promote caregivers’ positive feelings by utilizing relevant mindfulness materials, which could then be verified. In summary, whether a mindfulness intervention acts as a direct influencing factor may depend on two different contexts or situations and the application of intervention materials needs to be clarified appropriately, both in clinical and differential research.

A growing number of applications and theories have shown that mindfulness training has a positive impact on participants, including positive thinking, emotions, and behaviors. Some examples are the positive effects on perceived stress [41], students’ physical aggression [42], and self-regulation [43]. In the field of theoretical research, many new creative theories are based on Langerian mindfulness theory. For instance, the monitor and acceptance theory [44], regarding the mechanisms of mindfulness training, has been used successfully to promote self-acceptance. Based on the above analysis, previous applications of mindfulness training somewhat supported our hypothesis that mindfulness training can enhance positive feelings in family caregivers, and not only relieve negative emotions as shown in earlier research. Thus, the current research is a step forward in adopting mindfulness learning for the cultivation of positive feelings and in expanding the application of mindfulness in the clinical field. Therefore, it is necessary to further explore the positive elements in mindfulness interventions and expand their application in the field of positive feelings.

This is one of the few studies focusing on the influence of mindfulness on caregivers’ positive feelings. However, this research would not be rigorous enough if limitations were not acknowledged. The study lacked a long-term follow-up; thus, it was not possible to track the persistence of the effects of the intervention and its effectiveness could not be ensured. Moreover, this research did not examine the effect of the mindfulness intervention on the actual care provided to patients. Future studies should track and evaluate the effect of interventions and include the behaviors of participants to better understand the intervention effects. Furthermore, it would be useful to extend mindfulness interventions to other groups, such as patients with cancer, health professionals, and even the general population. In addition, LMS is usually used as a measure of trait mindfulness, thus an alternative manipulation check beside the LMS would be helpful for future studies. Additionally, given the concerns about the reliance on subjective assessment tools for mindfulness, it is recommended that a direct creativity-based tool such as Triangle Task be adopted in the future [45]. Overall, this research is a pilot study in the health care field, lending preliminary support to the claim that mindfulness training can promote positive feelings in caregivers of patients with cancer.

## 5. Conclusions

This study has demonstrated that a mindfulness intervention can be an important tool to promote positive feelings in caregivers of patients with cancer, and illustrates the function of mindfulness interventions in the field of health care. Future studies could examine the influence of mindfulness interventions at the behavior level and their practical application. Moreover, future research could broaden the effects of mindfulness interventions using other perspectives or multiple backgrounds.
